# Histogram-Based Calibration Method for Pipeline ADCs

**DOI:** 10.1371/journal.pone.0129736

**Published:** 2015-06-12

**Authors:** Hyeonuk Son, Jaewon Jang, Heetae Kim, Sungho Kang

**Affiliations:** Department of Electrical and Electronics Engineering, Yonsei University, 120-749 Seodaemoon-Gu Shinchon-Dong 134 Seoul, Korea; University of California Berkeley, UNITED STATES

## Abstract

Measurement and calibration of an analog-to-digital converter (ADC) using a histogram-based method requires a large volume of data and a long test duration, especially for a high resolution ADC. A fast and accurate calibration method for pipelined ADCs is proposed in this research. The proposed calibration method composes histograms through the outputs of each stage and calculates error sources. The digitized outputs of a stage are influenced directly by the operation of the prior stage, so the results of the histogram provide the information of errors in the prior stage. The composed histograms reduce the required samples and thus calibration time being implemented by simple modules. For 14-bit resolution pipelined ADC, the measured maximum integral non-linearity (INL) is improved from 6.78 to 0.52 LSB, and the spurious-free dynamic range (SFDR) and signal-to-noise-and-distortion ratio (SNDR) are improved from 67.0 to 106.2dB and from 65.6 to 84.8dB, respectively.

## Introduction

Analog-to-digital converters (ADCs) which perform signal transfers between the analog and digital domains are considered as the most important devices in systems-on-chips (SoCs) [1]–[3]. ADCs have been widely used in applications such as communications, energy, healthcare, instrumentation and measurement, automotive, aerospace, and so on. Generally, ADCs are the largest bottleneck in the process of data transmission, indicating that an exact operation should be guaranteed. Especially, when they are used in healthcare SoCs or hybrid electronic vehicles [4, 5], a minor malfunction can cause a fatal accident. In addition, as the clock speed of SoCs increases in 3D-ICs including TSVs(Through-Silicon Vias), ADCs with the higher speed have been required. For these reasons, calibration methods for accuracy and specification have been applied in many ADCs.

Since pipelined ADCs can operate with high-speed and high-resolution simultaneously, they are widely used in the domain that successive approximation ADCs and time-interleaving ADCs cannot cover [6]. However, pipelined ADCs inevitably include capacitor mismatch and finite op-amp gain in each stage due to their structure, which generates a non-linearity error so that a calibration technique is required for improved performance [7, 8]. The digital calibration technique needs less power and less hardware overhead and improves performance more as CMOS technology improves, so they have been researched in many studies [8]–[13].

Previous digital calibration methods have measured the non-linearity characteristic of the error sources that occur in multiplying DACs (MDACs) and have applied the inverse transfer function to it. In [8], an interpolation-based calibration method is introduced to compensate both linear and nonlinear errors from pipeline stages. In this method, a parallel calibration ADC channel is used to prevent the propagation of errors. It has an advantage that requires little calibration overhead and short convergence time. However, the calibration technique in [8] has too complex procedures to get the optimal solution. The calibration method proposed in [9] improves the linearity of two-step ADC by simultaneously subtracting switch feedthrough, op-amp offset and inter-stage gain from the digital outputs. This method can be implemented simply using only some extra digital logic, which does not influence the original structure. However, it does not consider the finite op-amp gain errors which seriously impact on the performance of pipelined ADCs. The background calibration method described in [13] estimates the inter-stage gain of each stage by conventional foreground calibration technique. An initial estimation of inter-stage gain is conducted in foreground, and then the gain variations due to temperature and voltage are tracked in background. This previous method has an advantage of short calibration time but low accuracy of calibration results is a weakness.

In this research, a histogram-based digital calibration technique for pipelined ADCs is proposed. The proposed method targets capacitor mismatches and finite op-amp gain errors of MDACs in each pipeline stage. To reduce the calibration time without the loss of performance, it generates stage-histograms through the digitized outputs of each stage and calibrates the errors by analyzing the histograms. The stage-histograms and additional calibration modules can be implemented with low hardware overhead. After the proposed calibration method, significant linearity improvements can be obtained with fewer calibration times compared with the previous methods.

### Background

**Fig 1:**
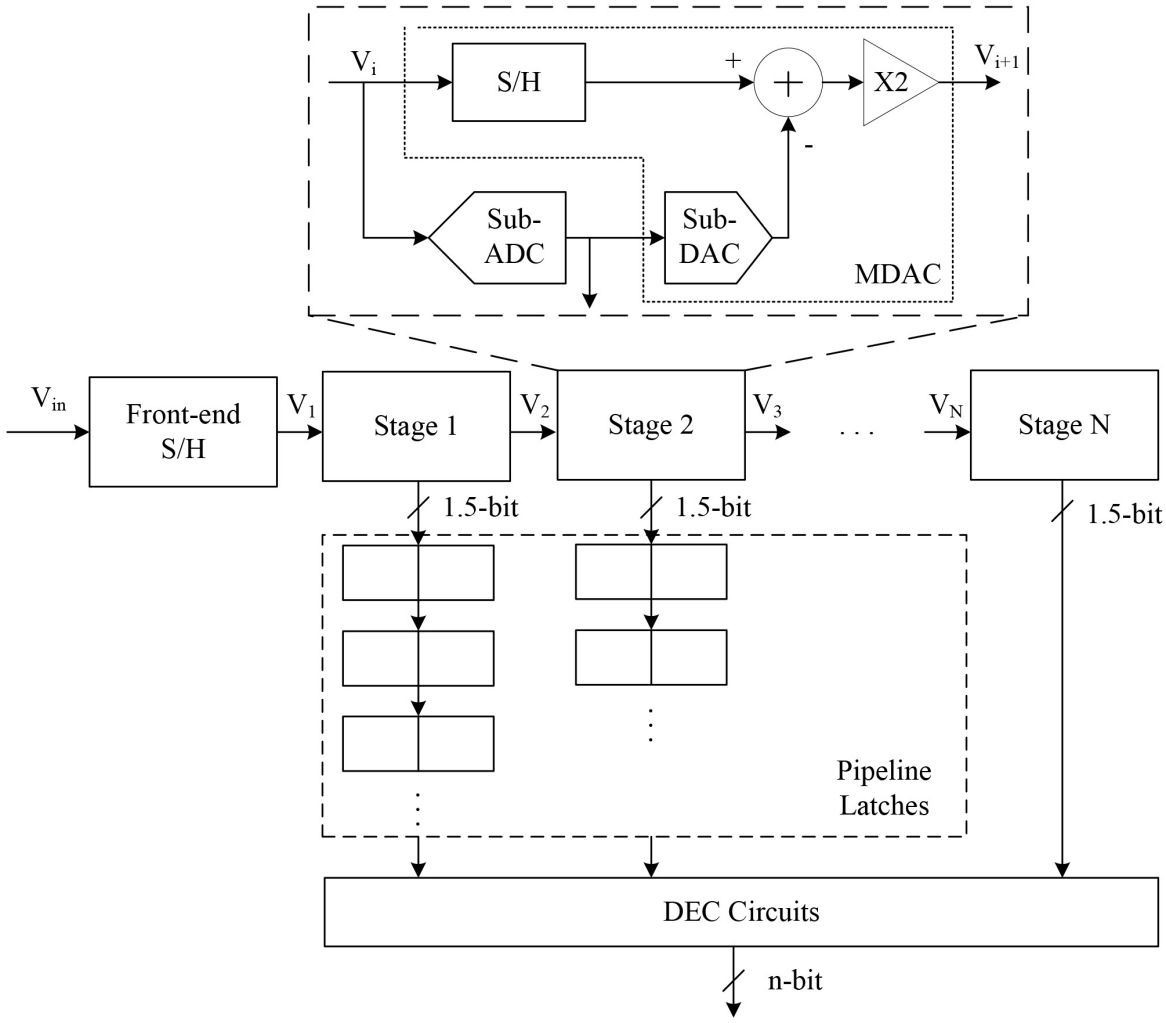
General architecture of a 1.5-bit/stage pipelined ADC.

The general structure of a 1.5-bit/stage pipelined ADC is shown in Fig 1 [14]. As described in Fig 1, a pipelined ADC includes front-end S/H circuits and N stages arranged in series. Each stage consists of an S/H circuit, sub-ADC, sub-DAC, subtractor, and gain stage. The output (V_i_) of the previous stage goes through the S/H circuits and the sub-ADC generating the pipeline stage outputs. Simultaneously, the residues are produced through the sub-DAC, subtractor, and gain stage and delivered to the next stage. The outputs occurring at each stage are stored in the pipeline latches and go through the DEC circuits. After traversing the DEC circuits, the final outputs are determined, eliminating some errors. The DEC circuits perform digital correction using multiple bits occurring at each stage. The multi-bit pipeline stages reward some comparator offset errors by setting the ‘01’ stage between output ‘00’ and ‘10’ compared to single-bit pipelined stages that generate an output of ‘0’ or ‘1’ at each stage. The threshold voltage is generally set to ±1/4 V_ref_, and the more accurate outputs can then be obtained.

## Method

### Error analysis on inter-stages

**Fig 2:**
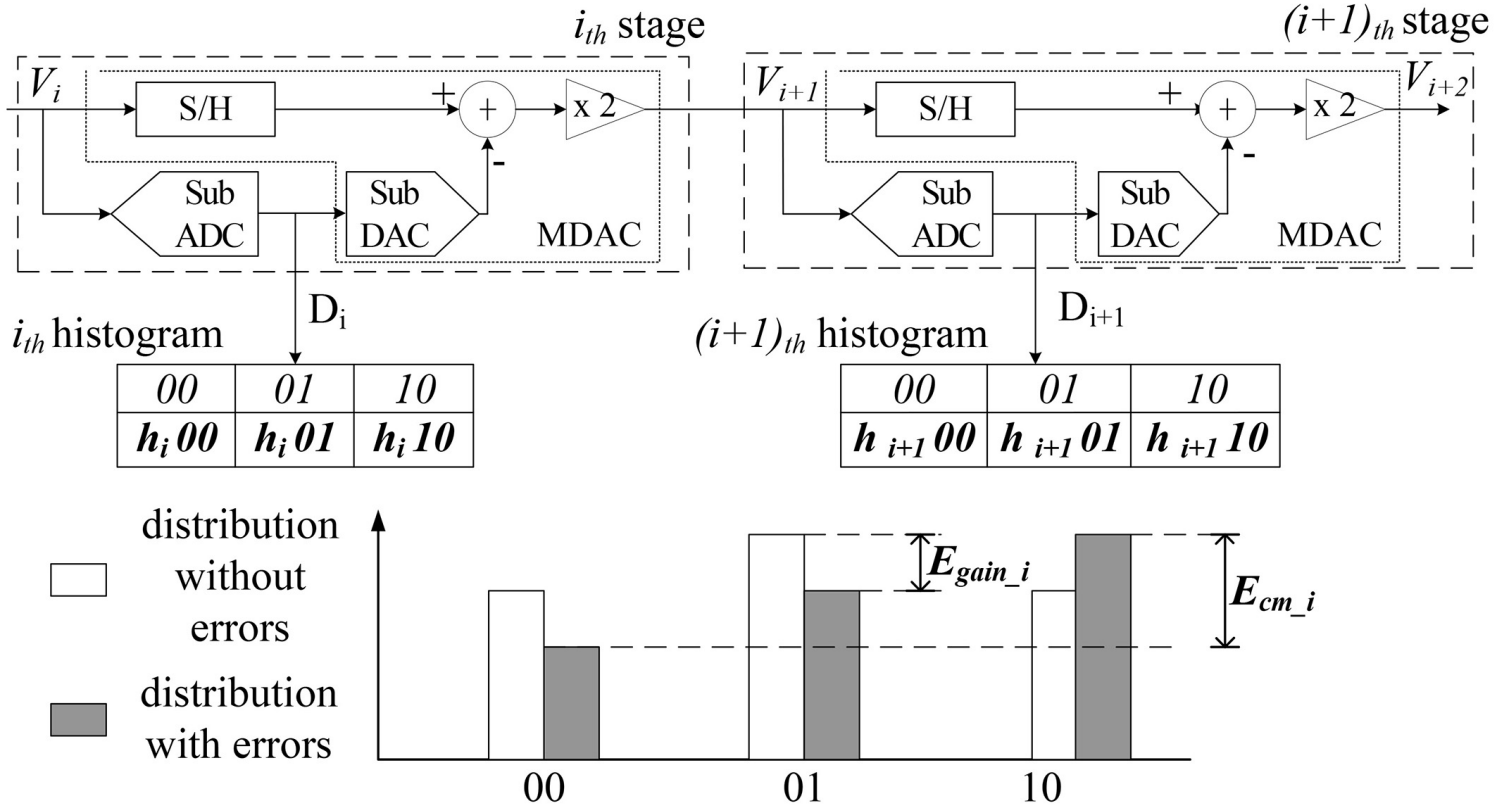
The proposed calibration method based on stage-histogram data.

A notation table to summarize the following key variables is shown in Table 1. The proposed calibration method composes stage-histograms using the outputs of each stage and determines the error sources by analyzing these histograms. The proposed calibration method targets non-linearity errors due to capacitor mismatches and finite op-amp gains of MDACs. Fig 2 shows the proposed calibration method based on stage-histogram data. The errors in MDACs change the distribution of the outputs that occur in the process of digitizing through the sub-ADC of the next stage. The transfer function at the i_th_ stage, including the capacitor mismatch (E_cm i_) and gain error (E_gain i_), is decided as shown in (1). In this research, the proposed method is explained based on a 1.5-bit/stage pipelined ADC, but it can be applied in another multiple-bit/stage structure, using the same principle.

$$\begin{array}{c} y=\left(2+E_{gaini}\right)\left(x+k\cdot V_{ref}/2\right)+E_{cmi}\left(k=-1,0,1\right) \end{array}$$ (1)

**Table 1:** Notation of the following key variables.

E_cm i_	Capacitor mismatch error at i_th_ stage
E_gain i_	Gain error at i_th_ stage
D_i_	Digital output of i_th_ stage
P(D_i_ = y)	Probability that D_i_ is equal to y (y = 00, 01, 10)

**Fig 3:**
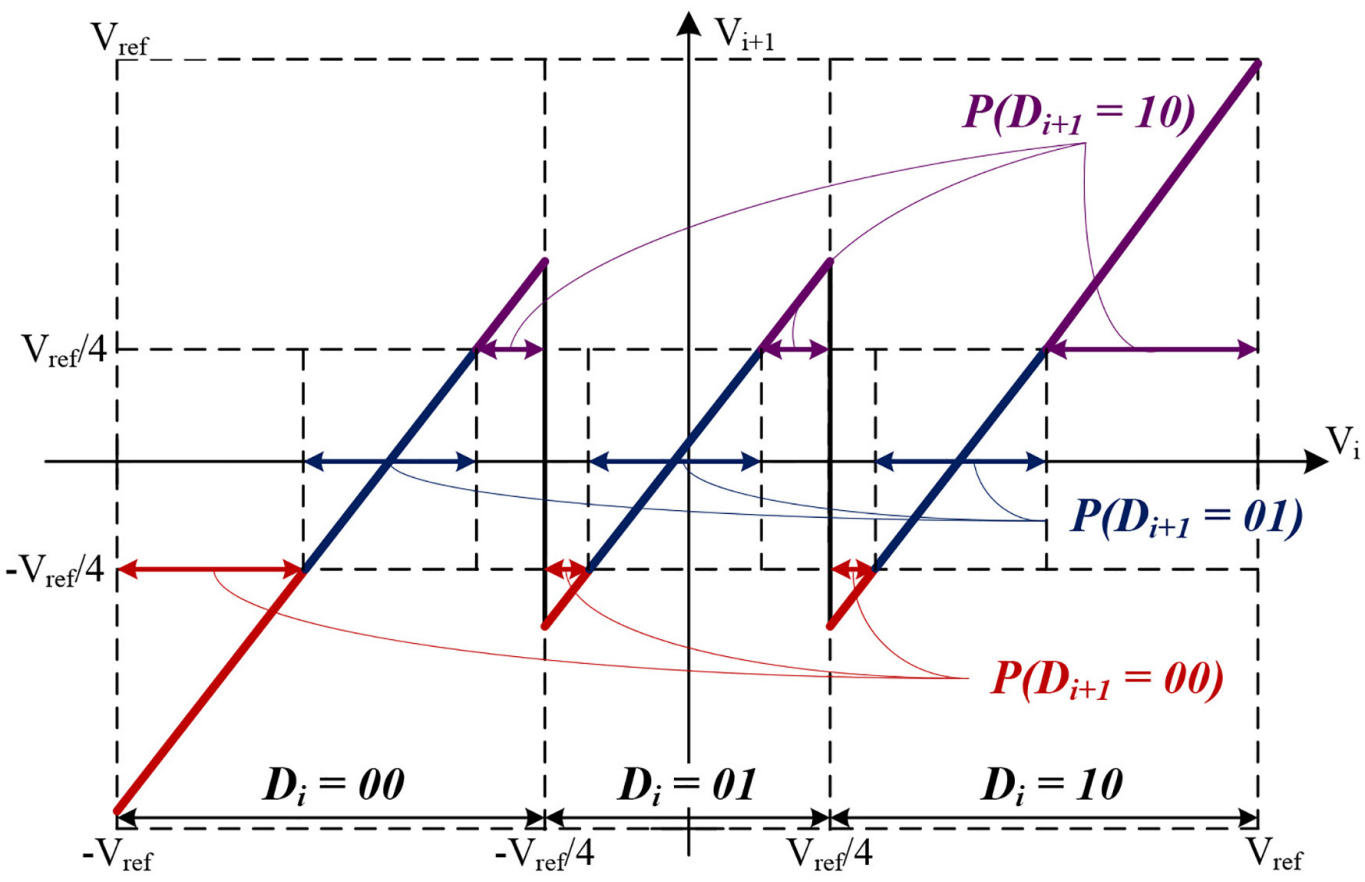
The transfer function including non-linearity errors and the output distribution in the next stage.

The digitized outputs of the (i+1)_th_ stage(D_i+1_) are influenced by the operation of i_th_ stage, so the results of (i+1)_th_ histogram reflects the errors in i_th_ stage. The effects of E_cm i_ and E_gain i_ are derived by the collected histogram data. To determine the effects of inserted errors on histograms, the range of each outputs in the next stage are calculated. The transition points, where the output values change, can be obtained by measuring *x* when substituting v_ref_/4 for *y* in (1). Fig 3 shows the output ranges on the transfer curve including non-linearity errors.

The length of each section where the values of 00, 01, and 10 in the next stage are calculated and the results are expressed as (2). We assume that the input distribution of each stage is uniform. From (2), a capacitor mismatch which raises or lowers the outputs can be determined by the difference between the 10 and 00 probability values (p(D_i+1_ = 10) from p(D_i+1_ = 00)). In addition, a gain error changes the slope of the output transfer curve of the next stage, which can be calculated through the probability of the 01 value that occurs in the next stage. The determining error sources can be written as (3). The *α* in (3) are determined by the result of E_gain i_ and the *β* and *γ* values are decided by the specifications of the ADC, such as the conversion rate, input range. The calculated error sources are applied to the calibrated outputs during the DEC process for eliminating non-linearities.

$$\begin{array}{ccc} p(D_{i+1}=00) & = & \frac{\left(5+4\cdot E_{gain}\right)V_{ref}-12E_{cm}}{8+4E_{gain}}\\ p(D_{i+1}=01) & = & \frac{6V_{ref}}{8+4E_{gain}}\\ p(D_{i+1}=10) & = & \frac{\left(5+4\cdot E_{gain}\right)V_{ref}+12E_{cm}}{8+4E_{gain}} \end{array}$$ (2)

$$\begin{array}{ccc} E_{cmi} & = & \alpha (p\left(D_{i+1}=10\right)-p\left(D_{i+1}=00\right))\\ E_{gaini} & = & \frac{\beta }{p(D_{i+1}=01)}-\gamma \end{array}$$ (3)

### Flow and structure of the histogram-based calibration

**Fig 4:**
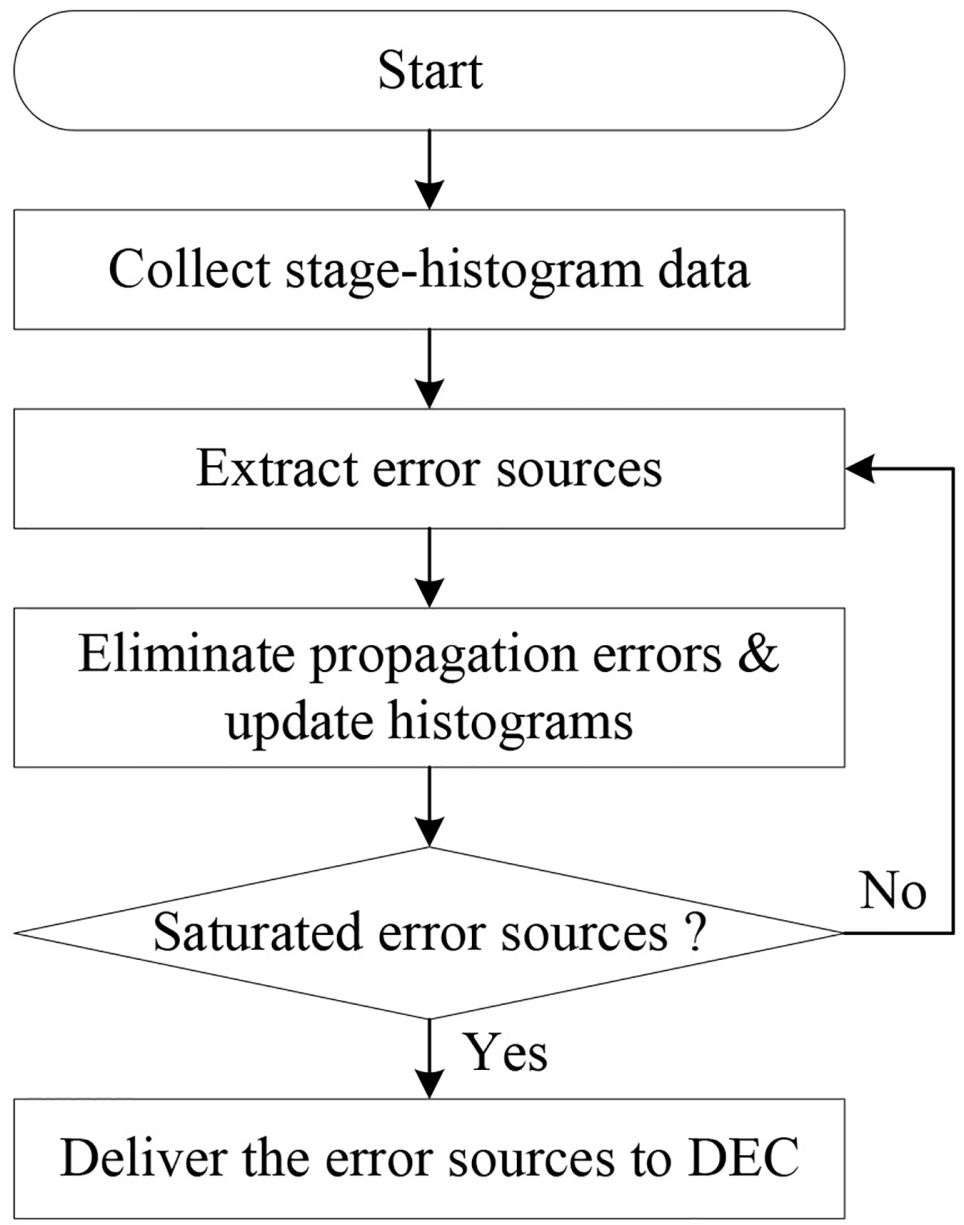
Flow chart of the proposed calibration method.

The flow chart of the proposed calibration is shown in Fig 4. The proposed method creates the stage-histograms and collects the fault information of each stage. When the enough samples are applied, the errors can be calculated by mathematical analysis above mentioned. The proposed calibration technique executes the propagation error elimination for more accurate results. The error sources generated in a stage affect the later stages in a specific ratio. The proposed calibration calculates the ratio and eliminates the propagation errors updating the histogram data. These procedures are repeated from the first stage to the last stage and the error sources are extracted when the histogram data are saturated.

**Fig 5:**
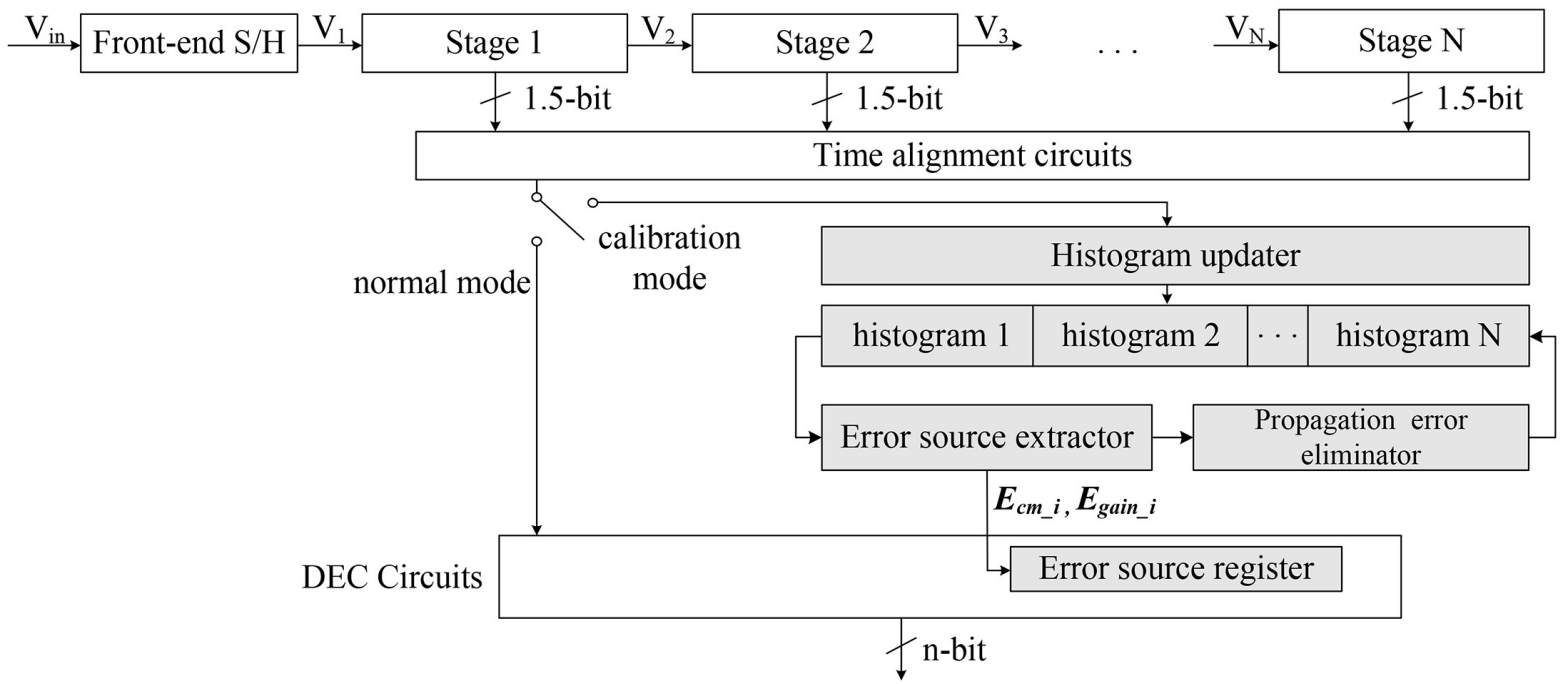
Pipelined ADC architecture with the proposed calibration method.

Fig 5 shows the hardware structure for the proposed calibration method. The circuits for the proposed digital calibration technique does not influence the operation of the original pipelined ADC. The histogram updater composes the stage-histogram until the sufficient samples are collected. If the data are inserted to the pipeline latch, the enable signal of the relevant line occurs according to the 00, 01, and 10. The error source extractor calculates the error sources and the propagation error eliminator apply them to the histograms. After the process, the error source extractor determines the information of error sources through the final histogram data and delivers them to DEC.

The proposed calibration architecture has an advantage to have low complexity in digital hardware. Since the stage-histograms are composed at each stage, the proposed method has significantly less area overhead than the conventional histogram method. The conventional histogram requires 2^N^ histogram data to distinguish the all cases of N-bit ADC [15], but the proposed method needs only 3N histogram data. The stage-histogram consists of several gates and counters to distinguish the 00, 01, and 10 values. The error source register in DEC also needs low area overhead because the compensation data are produced by the combination of the error sources in each stage.

## Results and Discussion

**Fig 6:**
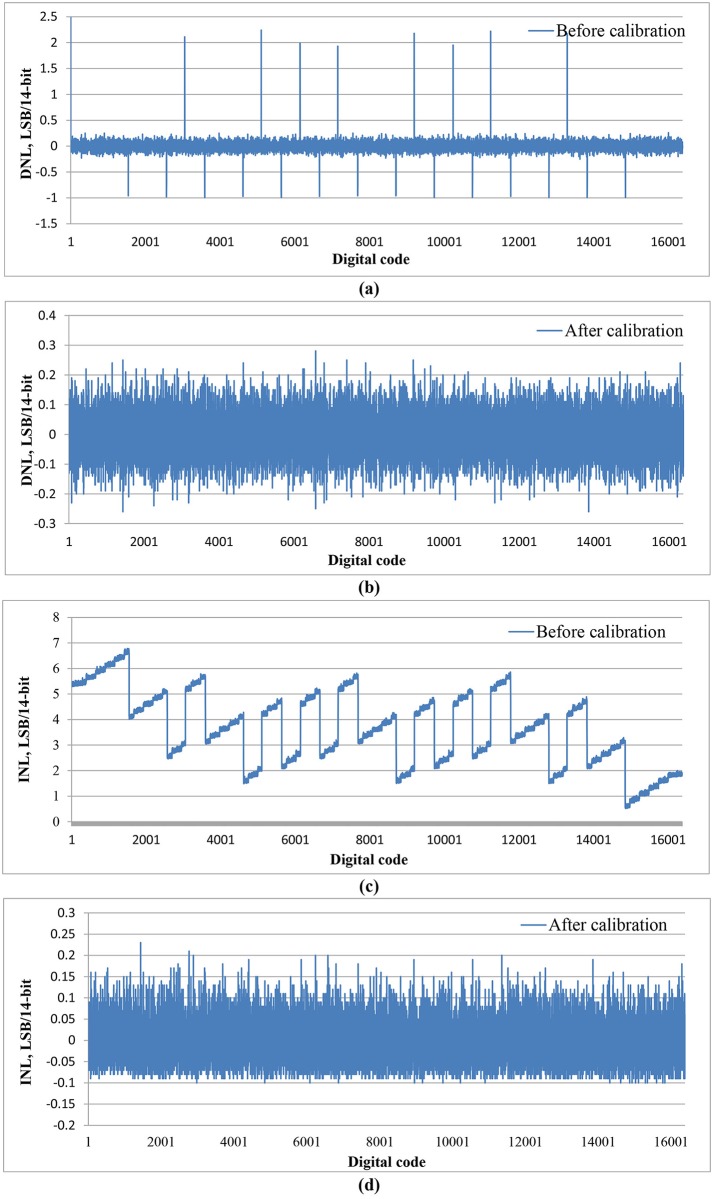
Results on static parameters before and after calibration. (a) Measured DNL errors before calibration (Maximum error: 5.44 LSB) (b) Measured DNL errors after calibration (Maximum error: 0.28 LSB) (c) Measured INL errors before calibration (Maximum error: 6.78 LSB) (d) Measured INL errors after calibration (Maximum error: 0.52 LSB)

**Fig 7:**
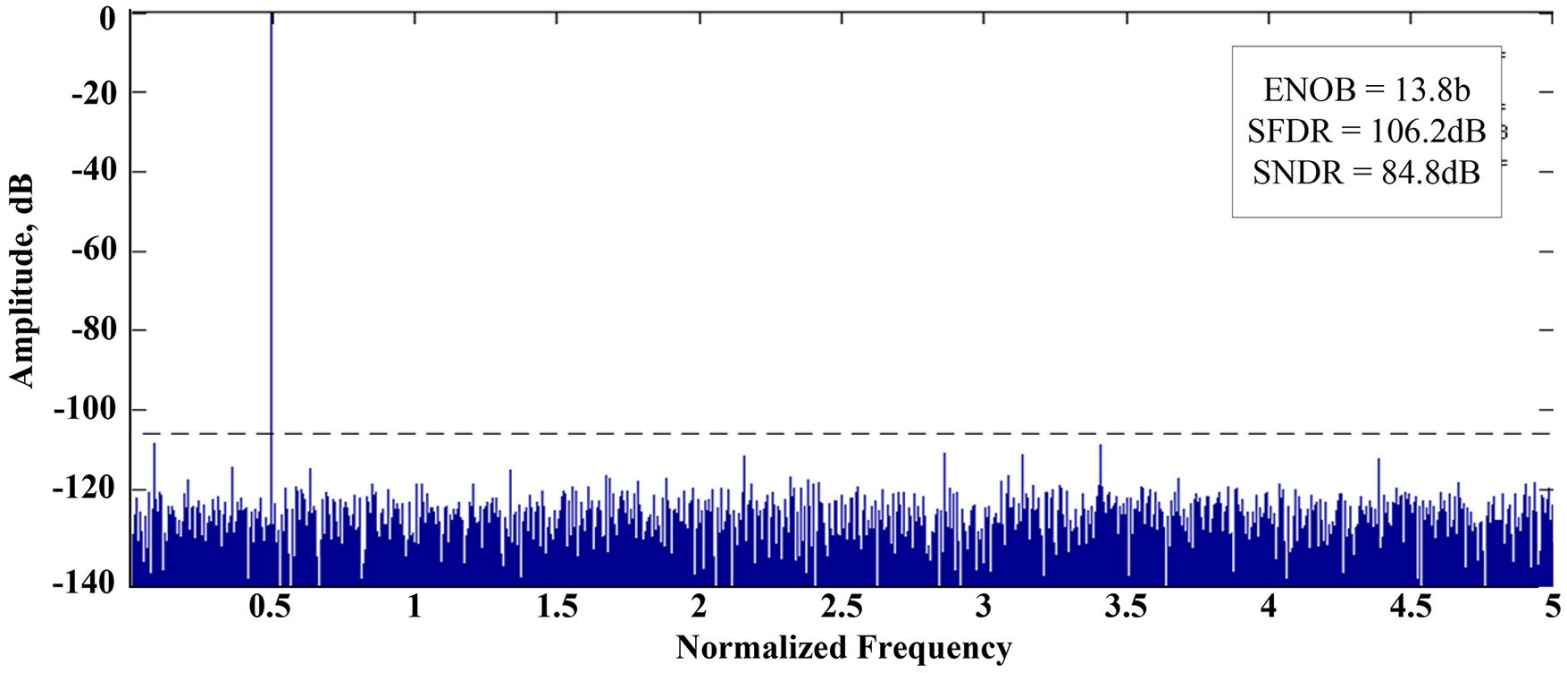
Frequency response after calibration.

MATLAB and C++ simulations were performed to prove the performance of the proposed calibration method. The simulations were performed on 14-bit pipelined ADCs with parameters in Table 2. The simulations were performed repetitively according to the various errors, and the maximum values were measured. The measured differential nonlinearity (DNL) and integral nonlinearity (INL) results before and after calibration are described in S1 File and Fig 6. The proposed calibration method improves the maximum DNL from 5.44 to 0.28 LSB and the INL from 6.78 to 0.52 LSB. Only 10 cycles of the input sequence are required to determine the static parameters. The results in terms of the effective number of bits (ENOB), spurious-free dynamic range (SFDR), and signal-to-noise-and-distortion ratio (SNDR) are plotted in Fig 7. After calibration, both the SFDR and SNDR are improved from 67.0 to 106.2 and from 65.6 to 84.8, respectively.

**Table 2:** Simulated parameters of pipelined ADC.

Reslolution	14-bit
Architecture	1.5-bit/stage
Swing	2V_p-p_
Capacitor mismatch	Maximum ± 0.3 LSB
Op-amp gain error	Maximum ± 0.2
Gaussian random noise	Mean: 0
	standard deviation: 0.001

**Fig 8:**
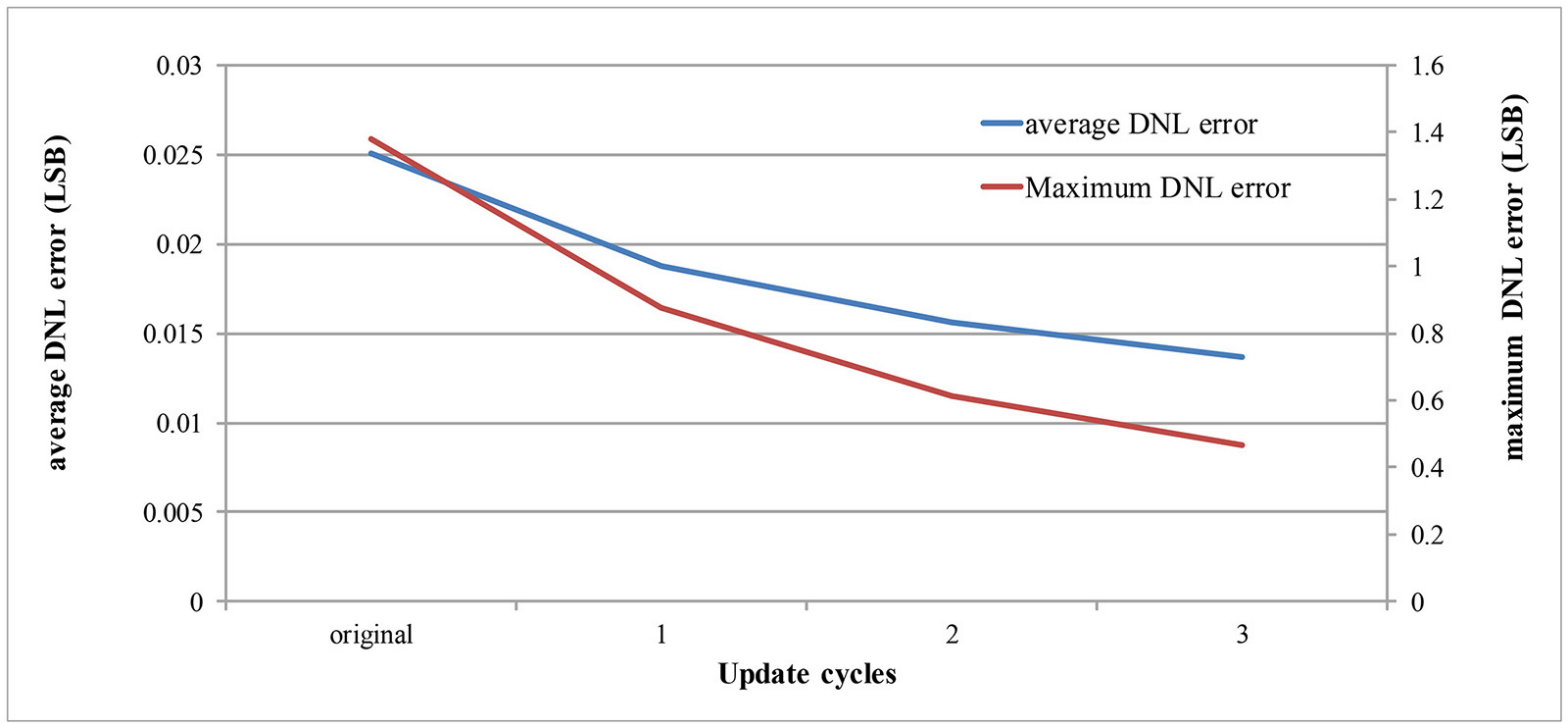
Average and maximum DNL errors in the process of histogram updates.

**Fig 9:**
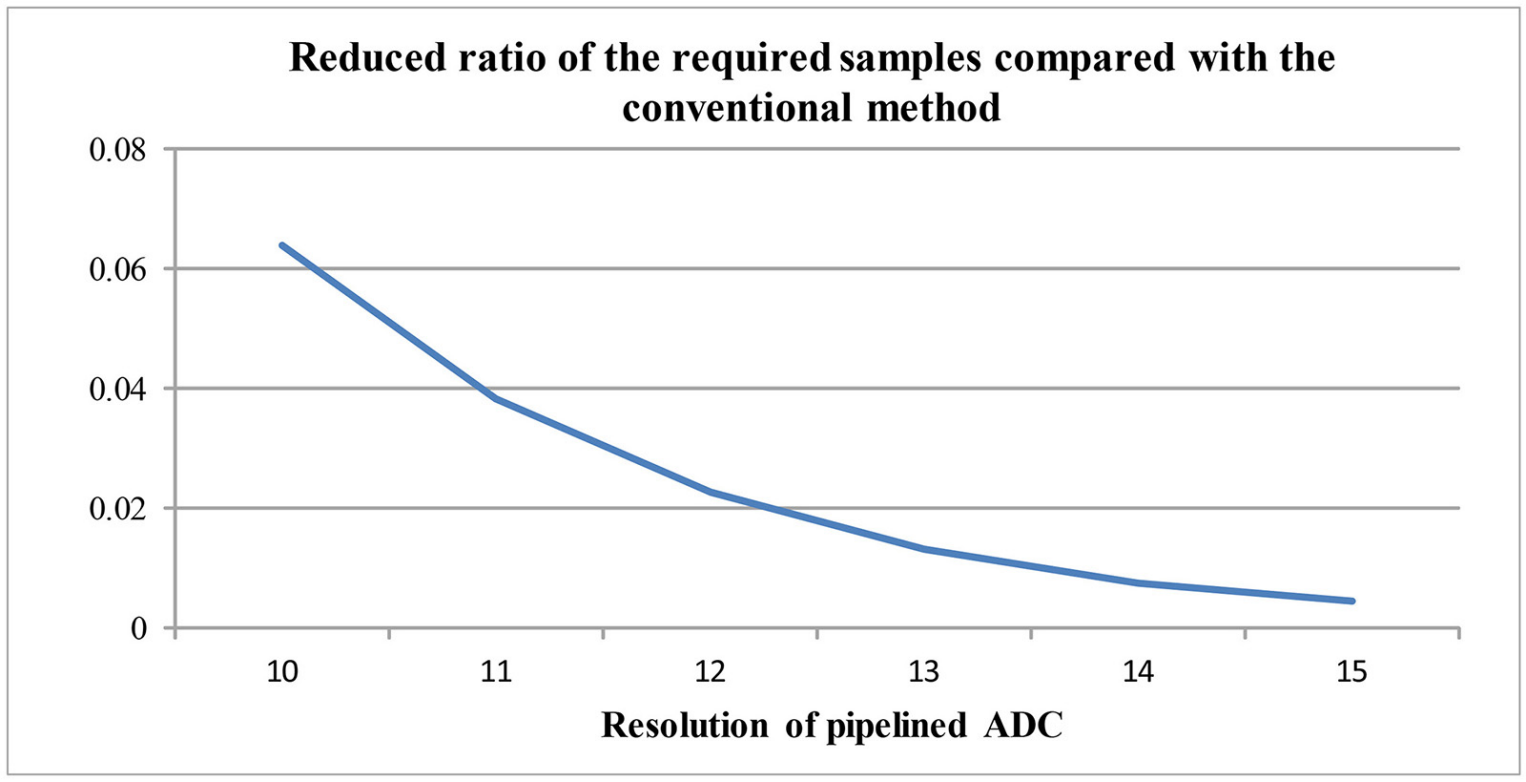
Ratio of the required input samples compared with the conventional histogram-based method.

S2 File and Fig 8 describes the average and maximum DNL errors when the histogram updates are progressed. In Fig 8, it shows that about 45% of average DNL errors and 66% of maximum DNL errors are reduced by the histogram updates of three cycles. S3 File and Fig 9 shows the ratio of the required input samples compared with the conventional histogram-based method. Since the proposed method calculates the errors using the stage-histogram, it requires about 1% samples compared with the conventional method.

**Fig 10:**
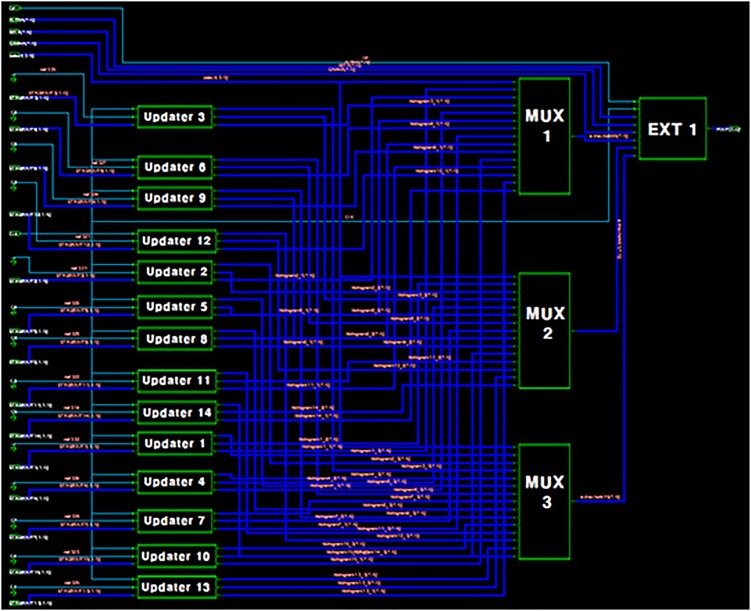
Synthesis results of the proposed architecture. Number of ports: 66, Number of nets: 440, Number of cells: 18.

To measure the area overhead caused by the proposed calibration method, Synopsys’s DesignVision was used to compile and synthesize the Verilog design. Fig 10 shows the synthesis results of the calibration circuits. From the provided area report, we can conclude that they require about 800 NAND gate counts. Compared with [8] which requires 912 gates only for decoders, the proposed method has the lower area overhead.

Table 3 shows the comparison between the proposed method and previous works. In the performance improvement, based on INL and SFDR, the proposed calibration method improves the performance from 6.78 to 0.52 LSB and from 65.6 to 84.8dB, respectively. Compared with [13] which improves SFDR from 69 to 85dB, the proposed method is more efficient in the calibration of non-linearity. In case of calibration time, [13] requires 2 million samples for 12-bit ADC and the proposed method requires 16 million samples for 14-bit ADC. Considering the resolution of ADC, the required calibration times can be considered as almost the same. Compared with [8], the proposed method shows performance enhancement on the same level as [8], but the low complexity of the hardware is the advantage of the proposed method compared with [8].

**Table 3:** Comparison with the previous works.

	J.Yuan [8]	C.Ravi [13]	Proposed method
Performance(in INL)	Accurate (from 7.85 to 0.27 LSB)	Less Accurate	Accurate (from 6.78 to 0.52 LSB)
Performance(in SFDR)	Accurate (from 52.5 to 84.4 dB)	Less Accurate (from 69 to 85 dB)	Accurate (from 67.0 to 106.2 dB)
Calibration time	Long	Short (about 2 million samples in 12-bit ADC)	Short (about 16 million samples in 14-bit ADC)
Hardware complexity	Medium	Low	Low

## Conclusions

This paper proposes a digital calibration method for pipelined ADCs based on stage-histogram data. The proposed calibration calculates the error sources in each stage by analyzing the stage-histogram and eliminates their effects in DEC circuits. Generating the histograms in each stage enables the accurate measurement of error sources, so the calibration time can be reduced. The implementation of the proposed method requires about 800 gate counts, which means the low hardware overhead compared with the previous works. In addition, it includes only digital process, so it does not modify the analog design of the ADCs. After calibration, the measured maximum INL is improved from 6.78 to 0.52 LSB, and the SFDR and SNDR are improved from 67.0 to 106.2dB and from 65.6 to 84.8dB, respectively.

## Supporting Information

S1 File Test results. Test results indluding DNL and INL in each step are described.
(XLSX)

S2 File Average and maximum DNL errors for proceeding update cycles. The reduction of DNL errors are shown as the proceeding update cycles.
(XLSX)

S3 File Reduced ratio of the required samples. The reduced ratio of the required samples for the proposed calibration method are calculated.
(XLSX)
